# Annotations

**Published:** 1893-01-07

**Authors:** 


### ANNOTATIONS.

Winters Ancient and Modern.

ARE we about to have "an old-fashioned winter"? It would appear so, if we may judge by the severity with which the season has already set in. But what was an "old fashioned winter" like? Accurate observations, unfortunately, were not very numerous; but Gilbert White—White of Selborne—was the most accurate of observers long before the days of generally accurate observation had even thought of dawning. Of the year 1768 White records that the frost in January was for some time the most severe that had then been known for many years. "The horses," he says, "fell sick with an epidemic distemper, which injured the winds of many and killed some." The disease was evidently pneumonia, due apparently entirely to the severe cold, and not to contagion. "Colds and coughs were general among the human species; meat was so hard that it could not be spitted; several redwings and thrushes were killed by the frost. To the great credit of Portugal laurels and American junipers," he continues, "be it remembered that they remained untouched amid the general havoc." The "havoc" being the complete destruction of many of Mr. White's most costly and highly-prized shrubs. In 1776, eight years later, a winter of equal or even greater severity was experienced. "Many of the narrow roads," in Surrey, "were now filled above the tops of the hedges, through which the snow was driven into the most romantic and grotesque shapes. The poultry dared not stir out of their roosting places, for cocks and hens are so dazzled and confounded by the glare of snow that they would soon perish without assistance. The hares also lay sullenly in their seats and would not move until compelled by hunger." A very unfortunate experience befel the fashionable ladies who crowded the city of Bath at that period for medicinal and other purposes. Those ladies wanted badly to go up to town for the Queen's birthday, which was to be celebrated on the 18th of the month. But "from the 14th," says the chronicler, "the snow continued to increase, and began to stop the road waggons and coaches, which could no longer keep on their regular stages. Many carriages of persons who got on their way to town from Bath as far as Marlborough, after strange embarrassments, here met with a *ne plus ultra*. The ladies"—poor ladies—"fretted, and offered large rewards to labourers if they would shovel them a track to London; but the relentless heaps of snow were too bulky to be removed; and so the 18th passed over, leaving the company in very uncomfortable circumstances at the 'Castle' and other inns." On the whole we seem to be a little better circumstanced for defying the severities of the seasons than our forefathers were. Our roads are better, and the railway lines can usually be promptly cleared from even the deepest snowdrifts. Whether our winters are, or are not, less severe cannot be satisfactorily determined. The probability is that they are not. But that we ourselves have made a considerable advance in power over our environment is very certain.

The Secret of Secrets.

THE departure of 1892 leaves us all a year older. Is the world, too, growing older, or is there the promise of immortal youth in it? Physiology has not solved the one problem which has exercised thinking men more deeply than any other during at least two thousand years—the problem of immortality. Whither is the race tending? Does science, in its profound explorations, come across any hint of future possibilites which may serve to hang a hope upon for the darkening days of old age? If science could but find a voice on this subject the world would listen with all its thousand million ears. In the meantime, it is plain that the most potent of all forces for the preservation of youthfulness of mind and body is this same hope of immortality which has laid hold upon so considerable a portion of the men and women of our struggling century. The *blasé* man who dismisses the teachings of the theologian as dreams and fables grows hopelessly, and often very painfully, old when once middle life has been passed. He has sucked the world dry, like an orange, and his power of enjoyment diminishes rapidly with the advancing years. A stoical endurance become his chief mainstay. On the other hand, the old-fashioned man, who is not turned from rooted convictions by passing phases of thought, visibly brightens up as age comes on. He has more confidence in the inner convictions of his being than in mere academic reasonings. After all, he thinks, the arguments for immortality are at least as weighty as those against it. When he adds his own convictions and hopes to the *pros* he finds that the *cons* speedily kick the beam. This man, who has a capacity for faith, and encourages it, grows younger with the dawn of each new year. He has discovered the secret which truly may be called the "secret of secrets," the secret of immortal youth. This thing is worthy of the consideration of men of science. The effect is obvious, and cannot be denied. Men who hold firmly to the belief in immortality have a happiness and youthfulness in old age which is denied to other men. The cause of this effect is worth searching out. Is it a real cause? Can there be a cause which is not real? Must not every real effect have a real cause, and an adequate cause? Physical science is losing a little of its paramount interest. Mental science will have its turn again in good time.

Medical Protection.

THE London and Counties' Medical Protection Society is making rapid headway. Last week we reported the formation of about 40 new branches in various parts of the country, and the adhesion of 140 new members. The Medical Defence Union on the other hand seems resolutely bent upon immediate suicide. It actually protests, in the *British Medical Journal*, against the publication, even in the medical newspapers, of those details of its proceedings which involve questions of fundamental policy. In other words it considers that the proper course for an association which desires to represent and to be of service to the whole medical profession, is to deny to the profession any knowledge whatever of its methods of working. This may be rational conduct from a Birmingham point of view; but from the point of view of useful business it is childish and ridiculous. Medical men are men and not children, whatever Mr. Lawson Tait and his admirers may think to the contrary. The truth is that the Medical Defence Union has been singularly unfortunate in its choice of officers. Impulsiveness, rashness, egotism, and an infinite capacity for fickleness of purpose, are not the characteristics of permanently successful leaders of men. These, nevertheless, seem to be conspicuous features at the Council Board of the Medical Defence Union. On the other hand, the Medical Protection Society has gone for men of a solid and sober temper of mind. Mr. Jonathan Hutchinson, its president, is known to all the profession as a judicial and judicious man; a man who weighs his actions with impartial justice, and who takes care that any association to which he gives his countenance and support shall be estimated by the same judicial scales. We have no desire to be unduly severe upon the Medical Defence Union; but it is the business of critics to be fair and to speak out with plainness. It is obvious that if the Union does not mend its ways, its days are numbered.

